# Exploring variations in IPC competencies: a cross-sectional study among healthcare professionals in Northwest China

**DOI:** 10.1186/s12879-024-09288-y

**Published:** 2024-04-22

**Authors:** Qinglan Zhao, Xiaoqing Cui, Ting Liu, Hanxue Li, Miaoyue Shi, Li Wang

**Affiliations:** 1 Infection Management Office, Xinjiang Uygur Autonomous Region People’s Hospital, Urumqi, Xinjiang China; 2 Nosocomial Infection Management Office, The Second Affiliated Hospital of Xi’an Jiaotong University, Xi’an, Shaanxi China; 3 College of Computer Science and Technology, Jilin University, Changchun, Jilin China

**Keywords:** Infection prevention and control (IPC), Healthcare professionals, Core competencies, Healthcare-associated infections

## Abstract

**Background:**

This cross-sectional study investigates infection prevention and control (IPC) competencies among healthcare professionals in northwest China, examining the influence of demographic factors, job titles, education, work experience, and hospital levels.

**Methods:**

Data from 874 respondents across 47 hospitals were collected through surveys assessing 16 major IPC domains. Statistical analyses, including Mann-Whitney tests, were employed to compare competencies across variables.

**Results:**

Significant differences were identified based on gender, job titles, education, work experience, and hospital levels. Females demonstrated higher IPC competencies, while senior positions exhibited superior performance. Higher educational attainment and prolonged work experience positively correlated with enhanced competencies. Variances across hospital levels underscored context-specific competencies.

**Conclusion:**

Demographic factors and professional variables significantly shape IPC competencies. Tailored training, considering gender differences and job roles, is crucial. Higher education and prolonged work experience positively impact proficiency. Context-specific interventions are essential for diverse hospital settings, informing strategies to enhance IPC skills and mitigate healthcare-associated infections effectively.

## Background

Healthcare-associated infections (HAIs) represent a persistent and concerning threat to patient safety, emphasizing the critical need for robust infection prevention and control (IPC) measures within healthcare settings [1, 2]. The prevalence of HAIs not only jeopardizes the well-being of patients but also poses challenges to the overall effectiveness and sustainability of healthcare systems [3]. In response to these challenges, healthcare institutions worldwide have established dedicated IPC teams comprising professionals with specialized knowledge and skills to design and implement effective strategies [4, 5].Effective IPC practices directly impact patient outcomes and overall healthcare quality. By reducing the incidence and transmission of HAIs, IPC measures play a pivotal role in safeguarding patient safety and improving healthcare outcomes.Moreover, effective IPC practices alleviate the burden of HAIs on healthcare systems. HAIs lead to increased morbidity and mortality rates, prolonged hospital stays, elevated healthcare costs, and resource strain. By preventing infections, healthcare facilities can enhance efficiency, optimize resource utilization, and elevate the quality of care provided to patients.The individuals at the forefront of these efforts, known as IPC specialists, play a pivotal role in preventing and controlling the spread of infections within medical facilities [6]. As the complexity of healthcare delivery continues to evolve, the role of IPC professionals becomes increasingly intricate. Their core competencies form the foundation for safeguarding patients, healthcare workers, and the broader community from the detrimental impact of healthcare-associated infections [7].

In the context of the COVID-19 pandemic, the imperative to enhance the core competencies of IPC professionals becomes even more pronounced [8–10]. These professionals must not only confront traditional infectious challenges but also adeptly respond to emerging threats such as the novel coronavirus. Therefore, continuous improvement in their knowledge base and skills, coupled with the ongoing refinement of infection control strategies, holds strategic importance in protecting patients, healthcare workers, preserving public health, and effectively responding to the spread of infectious diseases such as COVID-19 [11]. In this context, enhancing the core competencies of IPC professionals within healthcare institutions is an urgent and indispensable task [8].

Understanding the specific core competencies required by IPC specialists is essential for tailoring education and training programs that empower these professionals to meet the dynamic demands of their roles. The ability to effectively respond to emerging infectious threats, conduct thorough epidemiological investigations, implement stringent environmental monitoring, and educate healthcare personnel on best practices are just a few examples of the multifaceted skills demanded by this profession [12, 13].

This research endeavors to explore and delineate the essential core competencies of IPC specialists within healthcare institutions in Northwest China. By doing so, it aims to contribute valuable insights into the intricacies of their roles and responsibilities, paving the way for targeted training programs that address the specific needs of these professionals. The significance of this study lies in its potential to enhance the competency of IPC specialists, ultimately fortifying healthcare systems against the constant threat of healthcare-associated infections.

## Materials and methods

This cross-sectional study collected a total of 1021 questionnaire responses from 47 hospitals in the Northwestern region of China, spanning the period from November 2022 to November 2023. Of these, 874 responses with complete information were deemed valid. The questionnaire encompassed 16 major sections and 64 sub-items(among the 16 major sections, the maximum number of sub-items is 7, while the minimum number is 2.), covering critical domains such as Infection Prevention and Control (IPC) Project Management and Leadership, Architectural Environment of Medical Institutions, Basic Microbiology, Prevention of Antibiotic Resistance, Monitoring Healthcare-Associated Infections, Standard Precautions, Transmission-Based Precautions, Cleaning and Reprocessing of Medical Devices, Prevention of Catheter-Related Bloodstream Infections, Prevention of Catheter-Associated Urinary Tract Infections, Prevention of Surgical Site Infections, Prevention of Healthcare-Associated Pneumonia, Prevention and Management of Healthcare-Associated Infection Outbreaks, IPC Education and Training, Quality and Patient Safety, and Occupational Health.We referenced the “WHO Core Competencies of Infection Prevention and Control Practitioners” and tailored our core competency questionnaire to align with the specific circumstances in China.

Each item included a self-assessment of the individual’s current proficiency in the respective core competency. Scores ranged from 1 (completely unacquainted) to 4 (fully proficient), with partial and basic proficiency represented by scores of 2 and 3, respectively. The total score for each participant across the 16 major sections was then calculated. All participants provided informed consent, and the study obtained approval from the Medical Ethics Committee.

For general information, frequencies, and percentages were utilized. Collective performance in each core competency was described using mean and standard deviation (SD). Additionally, to account for uncertainty, 95% confidence intervals (95% CI) were calculated for each core competency. Comparative analyses were presented using mean and SD, and inter-group comparisons were conducted using the Mann-Whitney test (GraphPad Prism version 9). A significance level of *P* < 0.05 was considered indicative of statistical differences.

## Results

The participants in the survey had an average age of 38.29 ± 9.217 years, with an age range of 21 to 66 years. In terms of gender distribution, the results revealed that 10.65% were male, while 89.35% were female. Regarding professional positions, 28.84% were heads of Infection Control Departments, and 71.16% were staff members. Concerning professional titles, 44.39% held junior positions, 27.94% held intermediate positions, 16.48% held associate senior positions, and 11.20% held senior positions. Work experience varied, with 17.28% having ≤ 3 years, 10.42% having 3–6 years, 11.20% having 7–10 years, and 61.12% having ≥ 10 years. The participants had diverse professional backgrounds: 18.54% in clinical medicine, 61.01% in nursing, 12.13% in public health, 3.43% in preventive medicine, 1.49% in pharmacy, 3.09% in clinical laboratory science, and 0.34% in other fields. Educational backgrounds included 36.84% with diploma and below, 60.03% with a bachelor’s degree, and 3.09% with a master’s degree or higher. Regarding the duration of engagement in infection prevention and control work, 58.05% had ≤ 3 years, 19.33% had 3–6 years, 11.68% had 7–10 years, and 11.00% had ≥ 10 years. About participation in professional training during infection control work, 32.02% participated, while 67.98% did not. Lastly, with respect to hospital levels, 5.27% were from level 1 hospitals, 65.82% from level 2 hospitals, and 28.91% from level 3 hospitals (as shown in Table 1).

**Table 1:** General Information of Participants

Category	Characteristic	Number (*n*)	Percentage (%)
Gender			
	Male	93	10.65%
	Female	781	89.35%
Job Title			
	Junior	388	44.39%
	Intermediate	244	27.94%
	Associate Senior	144	16.48%
	Chief Senior	98	11.20%
Education Level			
	Junior College and Below	322	36.84%
	Bachelor’s Degree	525	60.03%
	Master’s Degree and Above	27	3.09%
Years of Engaging in Infection Prevention and Control Work			
	≤ 3	507	58.05%
	3–6	169	19.33%
	7–10	102	11.68%
	≥ 10	96	11.00%
Participation in Professional Training During Engaging in Infection Control Work			
	Yes	280	32.02%
	No	594	67.98%
Hospital Level			
	Level 1	46	5.27%
	Level 2	575	65.82%
	Level 3	253	28.91%

Infection prevention and control (IPC).

According to Table 2, the overall performance of core competencies among healthcare infection prevention and control professionals is as follows: IPC Project Management and Leadership received an average score of 15.92 ± 5.259, Architectural Environment of Medical Institutions scored 11.66 ± 3.647, Basic Microbiology scored 4.333 ± 1.435, Prevention of Antibiotic Resistance scored 8.671 ± 2.809, Monitoring Healthcare-Associated Infections scored 11.29 ± 3.60, Standard Precautions scored 9.992 ± 3.285, Transmission-Based Precautions scored 9.695 ± 3.110, Cleaning and Reprocessing of Medical Devices scored 7.561 ± 2.406, Prevention of Catheter-Related Bloodstream Infections scored 11.36 ± 4.172, Prevention of Catheter-Associated Urinary Tract Infections scored 9.663 ± 3.181, Prevention of Surgical Site Infections scored 11.32 ± 4.025, Prevention of Healthcare-Associated Pneumonia scored 11.24 ± 3.864, Prevention and Management of Healthcare-Associated Infection Outbreaks scored 4.790 ± 1.586, IPC Education and Training scored 6.351 ± 2.352, Quality and Patient Safety scored 6.454 ± 2.351, and Occupational Health scored 7.373 ± 2.332.

**Table 2:** Overall core competencies

Content	Mean ± SD	95% CI
IPC Project Management and Leadership	15.92 ± 5.259	15.56–16.28
Healthcare Facility Environment	11.66 ± 3.647	11.42–11.91
Basic Microbiology	4.333 ± 1.435	4.238–4.431
Prevention of Antimicrobial Resistance	8.671 ± 2.809	8.480–8.862
Surveillance of Healthcare-Associated Infections	11.29 ± 3.60	11.05–11.54
Standard Precautions	9.992 ± 3.285	9.768–10.22
Transmission-Based Precautions	9.695 ± 3.110	9.484–9.907
Cleaning and Reprocessing of Medical Devices	7.561 ± 2.406	7.397–7.724
Prevention of Catheter-Associated Bloodstream Infections	11.36 ± 4.172	11.07–11.64
Prevention of Catheter-Associated Urinary Tract Infections	9.663 ± 3.181	9.446–9.897
Prevention of Surgical Site Infections	11.32 ± 4.025	11.04–11.59
Prevention of Healthcare-Associated Pneumonia	11.24 ± 3.864	10.98–11.51
Prevention and Management of Healthcare-Associated Infection Outbreaks	4.790 ± 1.586	4.682–4.898
IPC Education and Training	6.351 ± 2.352	6.191–6.511
Quality and Patient Safety	6.454 ± 2.351	6.294–6.614
Occupational Health	7.373 ± 2.332	7.215–7.532

Infection prevention and control (IPC)

Gender differences in core competencies among healthcare professionals in infection prevention and control (IPC) were examined (Table 3). While no significant disparities were noted in several domains, notable variations surfaced. IPC Project Management and Leadership revealed a significant difference (*p* = 0.0126), favoring females (16.09 ± 5.249) over males (14.43 ± 5.069). Standard Precautions (*p* = 0.0374) and Transmission-Based Precautions (*p* = 0.0213) also favored females. Cleaning and Reprocessing of Medical Devices exhibited a significant gender gap (*p* < 0.0001). Females demonstrated higher scores in Prevention of Catheter-Related Bloodstream Infections (*p* = 0.0047), Prevention of Catheter-Associated Urinary Tract Infections (*p* < 0.0001), Prevention of Surgical Site Infections (*p* = 0.0003), and Prevention of Healthcare-Associated Pneumonia (*p* = 0.0013). IPC Education and Training (*p* = 0.0256), Quality and Patient Safety (*p* = 0.0485), and Occupational Health (*p* = 0.0359) also favored females. These findings underline gender-specific variations in IPC competencies, suggesting tailored training approaches for enhanced professional development.

**Table 3:** Gender Differences in Core Competencies

Content	Male Mean ± SD	Female Mean ± SD	*p*
IPC Project Management and Leadership	14.43 ± 5.069	16.09 ± 5.249	0.0126
Architectural Environment of Medical Institutions	11.03 ± 3.475	11.74 ± 3.662	0.0898
Basic Microbiology	4.165 ± 1.432	4.354 ± 1.434	0.2138
Prevention of Antibiotic Resistance	8.396 ± 2.992	8.692 ± 2.786	0.2279
Monitoring Healthcare-Associated Infections	11.04 ± 3.518	11.31 ± 3.612	0.5303
Standard Precautions	9.209 ± 2.935	10.08 ± 3.303	0.0374
Transmission-Based Precautions	9.000 ± 2.769	9.771 ± 3.127	0.0213
Cleaning and Reprocessing of Medical Devices	6.659 ± 1.851	7.668 ± 2.445	< 0.0001
Prevention of Catheter-Related Bloodstream Infections	10.12 ± 3.849	11.49 ± 4.179	0.0047
Prevention of Catheter-Associated Urinary Tract Infections	8.418 ± 2.813	9.777 ± 3.213	< 0.0001
Prevention of Surgical Site Infections	9.890 ± 3.650	11.45 ± 4.044	0.0003
Prevention of Healthcare-Associated Pneumonia	9.923 ± 3.260	11.40 ± 3.899	0.0013
Prevention and Management of Healthcare-Associated Infection Outbreaks	4.396 ± 1.444	4.841 ± 1.595	0.0059
IPC Education and Training	5.813 ± 1.949	6.420 ± 2.395	0.0256
Quality and Patient Safety	5.989 ± 2.052	6.520 ± 2.372	0.0485
Occupational Health	6.934 ± 2.097	7.428 ± 2.355	0.0359

Infection prevention and control (IPC)

Differences in core competencies across various job titles among healthcare professionals in infection prevention and control (IPC) were investigated (Table 4). Striking disparities emerged, highlighting the impact of job titles on competencies. All domains exhibited significant differences between Junior and Senior Associate positions (*p* < 0.0001). Senior Associates consistently outperformed their Junior counterparts. IPC Project Management and Leadership demonstrated a notable distinction. Similarly, the Architectural Environment of Healthcare Institutions, Basic Microbiology, Prevention of Antibiotic Resistance, Monitoring Healthcare-Associated Infections, Standard Precautions, Transmission-Based Precautions, Cleaning and Reprocessing of Medical Devices and Equipment, Prevention of Catheter-Related Bloodstream Infections, Prevention of Catheter-Associated Urinary Tract Infections, Prevention of Surgical Site Infections, Prevention of Healthcare-Associated Pneumonia, Prevention and Management of Healthcare-Associated Infection Outbreaks, IPC Education and Training, Quality and Patient Safety, and Occupational Health all displayed significant differences favoring Senior Associates. These findings underscore the influence of job titles on the acquisition and application of IPC core competencies, emphasizing the need for targeted training and professional development programs tailored to specific job roles.

**Table 4:** Differences in Job Titles for Core Competencies

Content	Junior Intermediate Mean ± SD	Senior Associate Mean ± SD	*p*
IPC Project Management and Leadership	14.95 ± 4.850	18.35 ± 5.438	< 0.0001
Architectural Environment of Healthcare Institutions	11.05 ± 3.324	13.24 ± 3.948	< 0.0001
Basic Microbiology	4.150 ± 1.382	4.788 ± 1.447	< 0.0001
Prevention of Antibiotic Resistance	8.249 ± 2.685	9.717 ± 2.845	< 0.0001
Monitoring Healthcare-Associated Infections	10.63 ± 3.352	12.92 ± 3.680	< 0.0001
Standard Precautions	9.374 ± 3.067	11.55 ± 3.270	< 0.0001
Transmission-Based Precautions	9.023 ± 2.810	11.38 ± 3.149	< 0.0001
Cleaning and Reprocessing of Medical Devices and Equipment	7.066 ± 2.220	8.808 ± 2.404	< 0.0001
Prevention of Catheter-Related Bloodstream Infections	10.43 ± 3.808	13.63 ± 4.150	< 0.0001
Prevention of Catheter-Associated Urinary Tract Infections	8.937 ± 2.965	11.40 ± 3.088	< 0.0001
Prevention of Surgical Site Infections	10.43 ± 3.729	13.45 ± 3.959	< 0.0001
Prevention of Healthcare-Associated Pneumonia	10.48 ± 3.551	13.14 ± 3.942	< 0.0001
Prevention and Management of Healthcare-Associated Infection Outbreaks	4.494 ± 1.494	5.542 ± 1.549	< 0.0001
IPC Education and Training	5.898 ± 2.174	7.513 ± 2.412	< 0.0001
Quality and Patient Safety	6.102 ± 2.227	7.379 ± 2.388	< 0.0001
Occupational Health	7.007 ± 2.201	8.304 ± 1	< 0.0001

Infection prevention and control (IPC)

Educational levels’ impact on the proficiency of infection prevention and control (IPC) core competencies among healthcare professionals was assessed, revealing significant differences (Table 5). Across all domains, individuals with a Bachelor’s degree and above consistently exhibited higher mean scores compared to those with a diploma and below (*p* < 0.05). These findings emphasize the positive association between higher educational attainment and enhanced proficiency in IPC core competencies, underscoring the importance of educational qualifications in shaping competency levels among healthcare professionals.

**Table 5:** Differences in Educational Levels for Core Competencies

Content	Diploma and below Mean ± SD	Bachelor’s and above Mean ± SD	*p*
IPC Project Management and Leadership	15.22 ± 5.382	16.32 ± 5.134	0.0028
Architectural Environment of Healthcare Institutions	11.29 ± 3.657	11.88 ± 3.627	0.0194
Basic Microbiology	4.116 ± 1.437	4.465 ± 1.418	0.0004
Prevention of Antibiotic Resistance	8.150 ± 2.826	8.966 ± 2.755	< 0.0001
Monitoring Healthcare-Associated Infections	10.75 ± 3.600	11.60 ± 3.567	0.0003
Standard Precautions	9.476 ± 3.087	10.29 ± 3.349	0.0003
Transmission-Based Precautions	9.194 ± 2.944	9.985 ± 3.153	0.0003
Cleaning and Reprocessing of Medical Devices and Equipment	7.216 ± 2.357	7.766 ± 2.417	0.0019
Prevention of Catheter-Related Bloodstream Infections	10.32 ± 4.007	11.95 ± 4.141	< 0.0001
Prevention of Catheter-Associated Urinary Tract Infections	8.868 ± 3.097	10.09 ± 3.173	< 0.0001
Prevention of Surgical Site Infections	10.22 ± 3.977	11.93 ± 3.929	< 0.0001
Prevention of Healthcare-Associated Pneumonia	10.54 ± 3.813	11.66 ± 3.832	< 0.0001
Prevention and Management of Healthcare-Associated Infection Outbreaks	4.552 ± 1.577	4.938 ± 1.573	0.0006
IPC Education and Training	6.125 ± 2.381	6.493 ± 2.335	0.0234
Quality and Patient Safety	6.245 ± 2.260	6.595 ± 2.385	0.0334
Occupational Health	7.047 ± 2.243	7.573 ± 2.365	0.0018

Infection prevention and control (IPC)

The impact of years of work experience on the proficiency of infection prevention and control (IPC) core competencies among healthcare professionals was explored, revealing substantial differences (Table 6). For each core competency, individuals with more than 6 years of work experience consistently demonstrated higher mean scores compared to those with 6 years and below (*p* < 0.0001). These findings underscore the positive association between longer professional experience and heightened proficiency in IPC core competencies, emphasizing the importance of accumulated work experience in shaping competency levels among healthcare professionals.

**Table 6:** Differences in Core Competencies by Years of Work Experience

Content	6 Years and Below Mean ± SD	6 Years Above Mean ± SD	*p*
IPC Project Management and Leadership	14.30 ± 4.555	18.13 ± 5.358	< 0.0001
Healthcare Facility Environment	10.59 ± 3.160	13.15 ± 3.772	< 0.0001
Basic Microbiology	4.039 ± 1.301	4.742 ± 1.514	< 0.0001
Prevention of Antimicrobial Resistance	7.973 ± 2.496	9.590 ± 2.951	< 0.0001
Surveillance of Healthcare-Associated Infections	10.24 ± 3.048	12.72 ± 3.820	< 0.0001
Standard Precautions	9.022 ± 2.895	11.31 ± 3.307	< 0.0001
Transmission-Based Precautions	8.679 ± 2.571	11.08 ± 3.220	< 0.0001
Cleaning and Reprocessing of Medical Devices	6.773 ± 2.108	8.654 ± 2.382	< 0.0001
Prevention of Catheter-Associated Bloodstream Infections	10.01 ± 3.472	13.16 ± 4.368	< 0.0001
Prevention of Catheter-Associated Urinary Tract Infections	8.579 ± 2.732	11.09 ± 3.240	< 0.0001
Prevention of Surgical Site Infections	9.961 ± 3.427	13.12 ± 4.103	< 0.0001
Prevention of Healthcare-Associated Pneumonia	9.994 ± 3.214	12.96 ± 4.041	< 0.0001
Prevention and Management of Healthcare-Associated Infection Outbreaks	4.311 ± 1.381	5.466 ± 1.612	< 0.0001
IPC Education and Training	5.714 ± 2.040	7.253 ± 2.486	< 0.0001
Quality and Patient Safety	5.863 ± 2.049	7.301 ± 2.481	< 0.0001
Occupational Health	6.810 ± 2.140	8.169 ± 2.371	< 0.0001

Infection prevention and control (IPC).

According to the results in Table 7, we found that all core competencies were higher among individuals with over 3 years of experience in infection prevention and control work compared to those with 3 years or less of experience. This trend was observed across various aspects ofIPC, including IPC project management and leadership, healthcare facility environment, basic microbiology, prevention of antimicrobial resistance, surveillance of healthcare-associated infections, standard precautions, transmission-based precautions, cleaning and reprocessing of medical devices, prevention of catheter-related bloodstream infections, prevention of catheter-associated urinary tract infections, prevention of surgical site infections, prevention of healthcare-associated pneumonia, prevention and management of healthcare-associated infection outbreaks, IPC education and training, quality and patient safety, and occupational health. These findings indicate that experienced professionals in infection prevention and control demonstrate higher scores across all core competencies, highlighting their proficiency in various aspects of IPC work.

**Table 7:** Differences in Core Competencies by Years of Engaging in Infection Prevention and Control Work

Content	3 Years and Below Mean ± SD	3 Years Above Mean ± SD	*p*
IPC Project Management and Leadership	14.29 ± 4.539	18.14 ± 5.356	< 0.0001
Healthcare Facility Environment	10.58 ± 3.148	13.15 ± 3.768	< 0.0001
Basic Microbiology	4.037 ± 1.297	4.745 ± 1.514	< 0.0001
Prevention of Antimicrobial Resistance	7.982 ± 2.493	9.579 ± 2.950	< 0.0001
Surveillance of Healthcare-Associated Infections	10.23 ± 3.036	12.73 ± 3.816	< 0.0001
Standard Precautions	9.014 ± 2.885	11.32 ± 3.312	< 0.0001
Transmission-Based Precautions	8.671 ± 2.562	11.09 ± 3.226	< 0.0001
Cleaning and Reprocessing of Medical Devices	6.767 ± 2.1	8.655 ± 2.379	< 0.0001
Prevention of Catheter-Associated Bloodstream Infections	10.02 ± 3.459	13.17 ± 4.363	< 0.0001
Prevention of Catheter-Associated Urinary Tract Infections	8.574 ± 2.722	11.09 ± 3.236	< 0.0001
Prevention of Surgical Site Infections	9.953 ± 3.414	13.12 ± 4.099	< 0.0001
Prevention of Healthcare-Associated Pneumonia	9.998 ± 3.202	12.95 ± 4.039	< 0.0001
Prevention and Management of Healthcare-Associated Infection Outbreaks	4.308 ± 1.376	5.462 ± 1.612	< 0.0001
IPC Education and Training	5.708 ± 2.032	7.249 ± 2.483	< 0.0001
Quality and Patient Safety	5.860 ± 2.042	7.297 ± 2.478	< 0.0001
Occupational Health	6.803 ± 2.133	8.165 ± 2.369	< 0.0001

Infection prevention and control (IPC).

The influence of participation in further education on the proficiency of infection prevention and control (IPC) core competencies was examined, highlighting significant differences across competencies (Table 8). Individuals who engaged in further education exhibited consistently higher mean scores compared to those who did not participate (*p* < 0.0001) across all core competencies. These findings underscore the positive association between active participation in further education and enhanced proficiency in IPC core competencies, emphasizing the importance of ongoing educational initiatives in maintaining and improving professional competency levels among healthcare practitioners.

**Table 8:** Differences in Core Competencies by Participation in Further Education

Content	During Further Education Mean ± SD	Not During Further Education Mean ± SD	*p*
IPC Project Management and Leadership	17.87 ± 5.145	15.01 ± 5.056	< 0.0001
Healthcare Facility Environment	13.00 ± 3.465	11.04 ± 3.565	< 0.0001
Basic Microbiology	4.649 ± 1.460	4.189 ± 1.400	< 0.0001
Prevention of Antimicrobial Resistance	9.470 ± 2.979	8.287 ± 2.646	< 0.0001
Surveillance of Healthcare-Associated Infections	12.29 ± 3.754	10.82 ± 3.434	< 0.0001
Standard Precautions	10.87 ± 3.130	9.576 ± 3.261	< 0.0001
Transmission-Based Precautions	10.54 ± 3.151	9.296 ± 2.995	< 0.0001
Cleaning and Reprocessing of Medical Devices	8.243 ± 2.343	7.246 ± 2.375	< 0.0001
Prevention of Catheter-Associated Bloodstream Infections	12.68 ± 4.144	10.73 ± 4.031	< 0.0001
Prevention of Catheter-Associated Urinary Tract Infections	10.57 ± 3.189	9.198 ± 3.111	< 0.0001
Prevention of Surgical Site Infections	12.50 ± 4.139	10.73 ± 3.857	< 0.0001
Prevention of Healthcare-Associated Pneumonia	12.52 ± 3.812	10.65 ± 3.741	< 0.0001
Prevention and Management of Healthcare-Associated Infection Outbreaks	5.228 ± 1.569	4.593 ± 1.553	< 0.0001
IPC Education and Training	7.239 ± 2.336	5.948 ± 2.256	< 0.0001
Quality and Patient Safety	7.190 ± 2.360	6.129 ± 2.261	< 0.0001
Occupational Health	8.011 ± 2.433	7.082 ± 2.226	< 0.0001

Infection prevention and control (IPC).

The investigation into variations in infection prevention and control (IPC) core competencies based on hospital level (Table 9) revealed significant differences across diverse competencies. In Level 1 and 2 Hospitals compared to Level 3 Hospitals, there were notable distinctions (*p* < 0.05) in IPC Project Management and Leadership, Healthcare Facility Environment, Basic Microbiology, Prevention of Antimicrobial Resistance, Surveillance of Healthcare-Associated Infections, Standard Precautions, Transmission-Based Precautions, Prevention of Catheter-Associated Bloodstream Infections, Prevention of Catheter-Associated Urinary Tract Infections, Prevention of Surgical Site Infections, Prevention of Healthcare-Associated Pneumonia, Prevention and Management of Healthcare-Associated Infection Outbreaks, IPC Education and Training, Quality and Patient Safety, and Occupational Health. This underscores the impact of hospital level on the proficiency of healthcare practitioners in various IPC core competencies. These findings can inform targeted interventions and educational programs tailored to specific hospital settings, contributing to a more effective and contextually relevant enhancement of IPC skills and knowledge.

**Table 9:** Differences in Core Competencies by Hospital Level

Content	Level 1 and 2 Hospitals Mean ± SD	Level 3 Hospitals Mean ± SD	*p*
IPC Project Management and Leadership	15.47 ± 5.227	17.08 ± 5.152	< 0.0001
Healthcare Facility Environment	11.36 ± 3.590	12.48 ± 3.679	0.0002
Basic Microbiology	4.184 ± 1.431	4.736 ± 1.369	< 0.0001
Prevention of Antimicrobial Resistance	8.320 ± 2.810	9.571 ± 2.596	< 0.0001
Surveillance of Healthcare-Associated Infections	10.87 ± 3.515	12.39 ± 3.606	< 0.0001
Standard Precautions	9.604 ± 3.148	11.00 ± 3.395	< 0.0001
Transmission-Based Precautions	9.389 ± 3.037	10.49 ± 3.125	< 0.0001
Cleaning and Reprocessing of Medical Devices	7.444 ± 2.373	7.870 ± 2.478	0.0534
Prevention of Catheter-Associated Bloodstream Infections	10.79 ± 4.069	12.82 ± 4.064	< 0.0001
Prevention of Catheter-Associated Urinary Tract Infections	9.281 ± 3.142	10.57 ± 3.165	< 0.0001
Prevention of Surgical Site Infections	10.78 ± 3.937	12.63 ± 3.979	< 0.0001
Prevention of Healthcare-Associated Pneumonia	10.74 ± 3.735	12.57 ± 3.883	< 0.0001
Prevention and Management of Healthcare-Associated Infection Outbreaks	4.701 ± 1.560	5.039 ± 1.627	0.0204
IPC Education and Training	6.124 ± 2.331	6.974 ± 2.322	< 0.0001
Quality and Patient Safety	6.315 ± 2.382	6.861 ± 2.196	0.0009
Occupational Health	7.165 ± 2.304	7.939 ± 2.319	< 0.0001

Infection prevention and control (IPC).

## Discussion

Our study, conducted in China’s northwest healthcare institutions, sheds light on the demographic dynamics influencing infection prevention and control (IPC) competencies among healthcare professionals in this region. The predominance of female participants (89.35%) and the representation of heads of Infection Control Departments (28.84%) underscore the need for gender-sensitive leadership programs. With a majority having over 10 years of experience (61.12%), the study reflects a seasoned workforce. However, the prevalence of nursing backgrounds (61.01%) signals a need for tailored training initiatives to accommodate diverse educational foundations within IPC. Varied engagement durations in IPC work and a significant portion (67.98%) not participating in professional training underscore the need for accessible and effective ongoing educational initiatives. Considering diverse experience levels and educational backgrounds in tailored training programs can comprehensively improve competencies. The distribution across hospital levels (65.82% from level 2 hospitals) emphasizes the regional perspective. Competency variations across hospital levels emphasize the need for context-specific training programs to address distinct challenges faced by healthcare professionals in different hospital settings within China’s northwest region.

The examination of gender differences in infection prevention and control (IPC) competencies among healthcare professionals in our study uncovers intriguing patterns. While several domains showed no significant disparities, notable variations emerged, emphasizing gender-specific nuances in IPC proficiency. Females exhibited superior scores in IPC Project Management and Leadership, Standard Precautions, Transmission-Based Precautions, Cleaning and Reprocessing of Medical Devices, and several infection prevention domains, indicating their enhanced competence in these critical areas. The observed gender-specific advantages highlight the need for tailored training programs acknowledging these differences. The significant gender gap in Cleaning and Reprocessing of Medical Devices suggests that female healthcare professionals excel in the intricacies of medical device sterilization and maintenance. Moreover, their higher scores in preventive measures against catheter-related infections and surgical site infections underscore their proficiency in ensuring patient safety during invasive procedures. The preference for females in IPC Education and Training, Quality and Patient Safety, and Occupational Health signifies their potential leadership in these realms. Research has shown that female physicians have outnumbered male participants in leading IPC programs [14]. Additionally, a study by P. Hlongwa indicates that females may have enhanced competence in some areas [15]. Furthermore, a study by Akan et al. found that the risk perception of males was significantly lower than that of females, indicating that females may have a better understanding of the risks associated with infection, which could contribute to their enhanced competence in IPC [16]. To optimize IPC competencies, healthcare institutions should recognize and leverage these gender-specific strengths, tailoring training initiatives to empower both male and female professionals effectively. This nuanced understanding contributes to fostering a diverse and skilled IPC workforce, ultimately enhancing healthcare outcomes.

Our investigation into the impact of job titles on infection prevention and control (IPC) competencies among healthcare professionals unveils substantial disparities, particularly between Junior and Senior Associate positions. The consistent outperformance of Senior Associates across all domains, including IPC Project Management and Leadership, Architectural Environment, Basic Microbiology, and various preventive measures, accentuates the pivotal influence of job roles on competency acquisition. This may be attributed to the self-perception and motivational abilities of individuals in higher-level positions, fostering IPC competence [17, 18]. This underscores the necessity for targeted training and development initiatives tailored to specific professional levels, ensuring a more nuanced and effective enhancement of IPC skills.

Moving to educational levels, our findings reveal a clear association between higher educational attainment and increased proficiency in IPC core competencies. Individuals with a Bachelor’s degree and above consistently exhibited higher mean scores across all domains compared to those with a diploma and below. This underscores the importance of educational qualifications in shaping the competency landscape among healthcare professionals in infection prevention and control. It has been found that clinical nurse educators with higher levels of education and greater lengths of work experience often report higher self-assessed levels of competence, highlighting the impact of educational backgrounds on competence levels [19].Institutions and policymakers should recognize the pivotal role of educational backgrounds, encouraging and facilitating continuous learning to ensure a skilled and competent IPC workforce capable of addressing evolving healthcare challenges.

The exploration of the impact of work experience on infection prevention and control (IPC) core competencies reveals a compelling association between longer professional tenure and heightened proficiency. Individuals with over 6 years of experience consistently demonstrated superior mean scores across all competencies, emphasizing the pivotal role of accumulated work experience in shaping the competency levels among healthcare professionals in IPC. Reeves et al. suggests that with more work experience, professionals are likely to have engaged in more IPE, thereby strengthening their IPC core competency [20]. This underscores the importance of recognizing and leveraging the expertise gained through years of practical engagement, advocating for continued professional development and mentorship.

Turning to the influence of further education, our investigation illuminates a positive correlation between active participation in ongoing educational initiatives and enhanced proficiency in IPC core competencies. Individuals engaged in further education consistently exhibited higher mean scores across all competencies, underscoring the crucial role of continuous learning in maintaining and elevating professional competency levels among healthcare practitioners in infection prevention and control. The European Centre for Disease Prevention and Control (ECDC)-commissioned Training in Infection Control in Europe project emphasizes the need for education in infection control and sets the stage for harmonization of IPC activities by issuing a list of core competencies for IPC professionals, further supporting the significance of educational qualifications in IPC [21]. Moreover, a study on interprofessional collaboration demonstrates that exposure to interprofessional education activities holds promise for enhancing IPC in clinical settings, emphasizing the role of education in promoting collaborative competence among healthcare professionals [22]. This emphasizes the imperative for healthcare institutions to facilitate and encourage access to educational opportunities, ensuring practitioners stay abreast of evolving best practices and advancements in IPC.

Analyzing variations based on hospital levels unravels noteworthy distinctions in IPC competencies. Level 1 and 2 Hospitals, compared to Level 3 Hospitals, exhibit significant differences across various competencies. This could be attributed to higher-level hospitals having a more comprehensive approach to the daily training of healthcare personnel and the setup of departments. It implies that higher-level hospitals possess stronger overall capabilities in responding to infectious diseases, enabling them to better handle various types of illnesses and medical situations [23]. This underscores the impact of the hospital level on the proficiency of healthcare practitioners, emphasizing the need for tailored interventions and educational programs catering to specific hospital settings. Such targeted approaches can contribute significantly to enhancing IPC skills and knowledge in a manner that is both effective and contextually relevant to the diverse healthcare landscapes within different hospital levels.

Despite the valuable insights gained, this study has limitations. Its cross-sectional design hinders establishing causation and observing changes over time. The focus on healthcare professionals in northwest China may limit generalizability to other regions. Self-assessment introduces social desirability bias, and the study lacks exploration of specific training programs. Additionally, while identifying differences in IPC competencies, it does not delve into the underlying reasons. Recognizing these limitations is crucial for interpreting findings and guiding future research efforts.

In conclusion, this study provides a comprehensive analysis of infection prevention and control competencies among healthcare professionals in northwest China. Demographic factors, job titles, education, work experience, and hospital levels significantly influence these competencies. Gender-specific variations and the impact of further education underscore the need for tailored training. Disparities between junior and senior positions highlight the importance of targeted professional development. Higher education positively correlates with enhanced proficiency. Longer work experience contributes to heightened competencies. Variances based on hospital levels emphasize the context-specific nature of IPC skills. Acknowledging these factors is vital for designing effective interventions and improving healthcare-associated infection prevention strategies.

## Data Availability

Data is provided within the manuscript.
